# Simultaneous bilateral midshaft clavicle fractures with unilateral dislocation of the acromioclavicular joint: A case report

**DOI:** 10.1097/MD.0000000000006975

**Published:** 2017-05-26

**Authors:** Dong Dong, Mingyang Yu, Guishan Gu

**Affiliations:** a Department of Radiology, First Hospital, Jilin University, Changchun, Jilin; b Department of Joint Surgery, Affiliated Zhongshan hospital, Dalian University, Dalian, Liaoning; c Department of Joint Surgery, First Hospital, Jilin University, Changchun, Jilin, China.

**Keywords:** dislocation of the acromioclavicular joint, mid-clavicle fracture, open reduction internal fixation

## Abstract

**Rationale:**

Simultaneous bilateral mid-clavicle fracture with unilateral dislocation of the acromioclavicular (AC) joint is an extremely rare injury combination.

**Patient concerns:**

Herein, we report a case of polytrauma in a 42 years old female following a road traffic accident.

**Diagnoses:**

The radiographs showed the fractures in bilateral middle third of the clavicle and AC joint dislocation of left shoulder.

**Interventions:**

The patient was treated with a 5-hole locking hook plate to stabilize the left AC joint and a shortly reconstruction plate to fix the left clavicle. Moreover, another 6-hole truncated anatomic reconstruction plate was used to fix the fracture of the right clavicle.

**Outcomes:**

At the 1-year follow-up, the patient had excellent range of motion and functional outcomes.

**Lessons:**

It is important to achieve stability and aim for excellence in terms of full shoulder function in this rare combination injury.

## 1 Introduction

About 70% to 80% of clavicle fractures occur in the middle third of the bone. However, a combined injury involving a fracture of the middle third of the clavicle and dislocation of the acromioclavicular (AC) joint is quite uncommon. Herein, we report a case of a female driver with simultaneous bilateral mid-clavicle fractures and unilateral dislocation of the AC joint. The surgical treatment used to manage the injury is described along with a discussion of the key learning points.

## 2 Case report

The study was approved by the ethics committees of the participating hospitals and institutes, and in accordance with the principles stated in the Declaration of Helsinki, and written informed consent was obtained from the patient.

**Figure 1:**
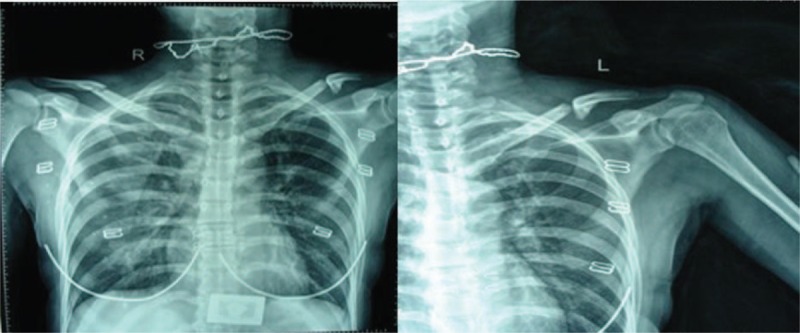
(A and B) Plain anteroposterior x-rays of the patient showing the rare combination injury in her left shoulder with isolated fracture of the middle third of the clavicle in the right shoulder.

**Figure 2:**
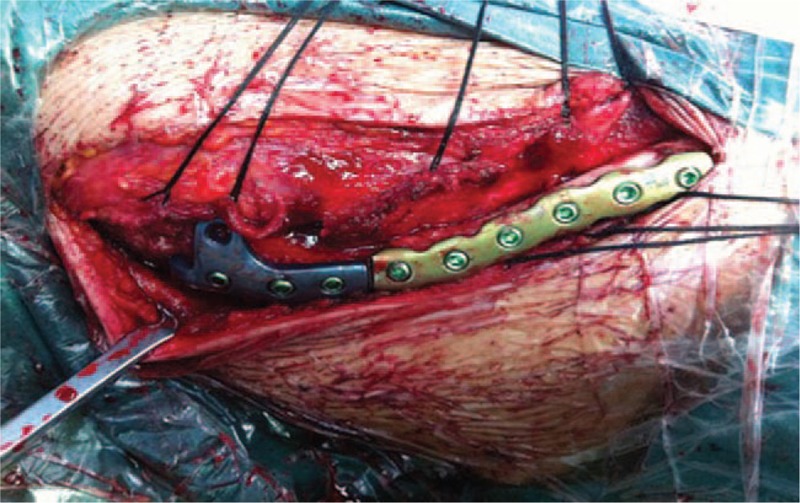
Intraoperative photograph showing combined fixation of locking hook plate and truncated anatomic reconstruction plate.

**Figure 3:**
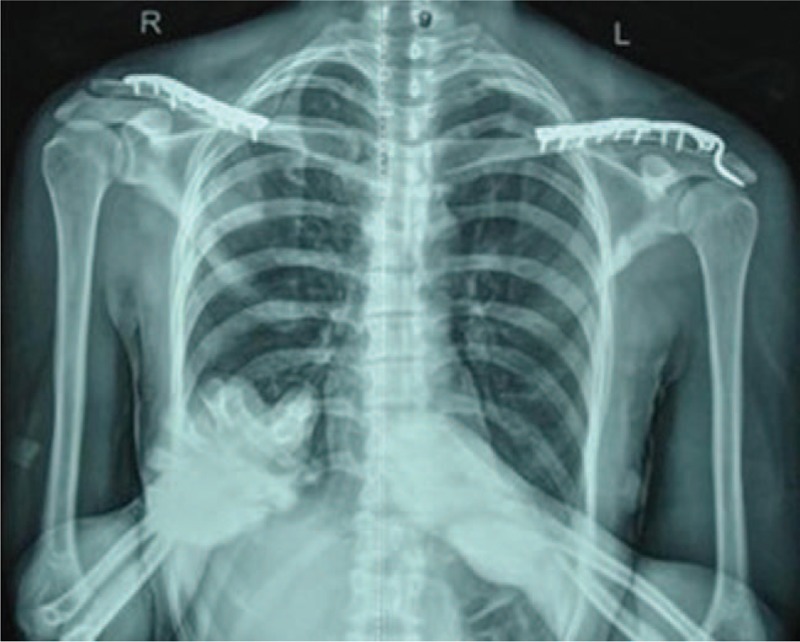
Postoperative radiograph showing the reconstructed clavicle with the truncated anatomic reconstruction plate and the acromioclavicular joint with the hook plate in place.

**Figure 4:**
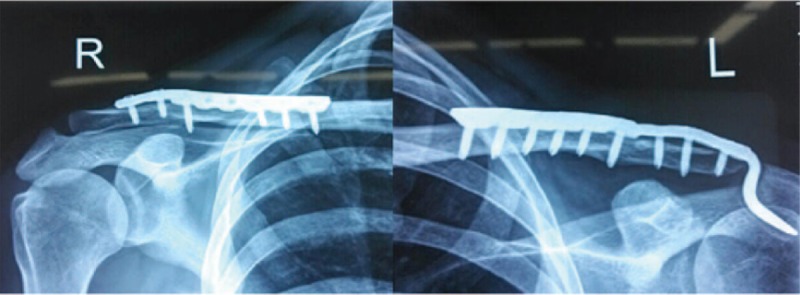
Radiograph taken at 1-year follow-up showing that the bilateral fractures had healed well. Clinically, the patient had excellent range of motion in both shoulders at 1 year.

A 42-year-old, left-hand-dominant female presented in the emergency department after being involved in a high-velocity road traffic crash. The patient was hemodynamically stabilized in the emergency department. Initial assessment revealed that she had contusion and swelling over the anterior aspect of the chest that extended up to the shoulders. At admission, the patient was taking shallow breaths and presented with pain during breathing, which was labored. The neurologic status and vascular status of the bilateral upper extremity were normal. Palpation revealed a palpable bony defect at the left acromion. A computed tomography scan of the thorax revealed a bilateral hemopneumothorax. The radiographs showed fractures in the bilateral middle third of the clavicle and AC joint dislocation in the left shoulder (Fig. 1). Given the nature of the injury, a decision to perform an operative stabilization was made. An incision in the skin of 15 to 16 cm in length was made from the AC joint to the medial end of the left clavicle. The soft tissues were incised and muscles were subperiosteally erased to expose the superior surface of the AC joint and the clavicle. Complete disruption of the AC and coracoclavicular (CC) ligaments was found, and the deltotrapezial fascia was stripped from its attachment to the distal clavicle. To stabilize the AC joint, we selected a 5-hole locking hook plate and placed it under the acromion and the AC joint. Another 6-hole truncated anatomic locking reconstruction plate was used to fix the fracture of the middle third of the left clavicle (Fig. 2). Contralateral surgery was performed at the same time. A similar approach was used to expose the clavicular fracture. A 6-cm incision was made over the superior aspect of the clavicle, centered over the fracture site. The subcutaneous tissue was incised and muscles subperiosteally erased. After achieving an anatomic reduction, an 8-hole locking compression plate was used to fix the fracture. Standard postoperative care was followed. Both shoulders were kept in a sling to ease pain. Postoperative radiographs revealed anatomical reduction of the fractures and stable reconstruction of both fractures and the AC joint (Fig. 3). Standard radiographs showed that the bilateral fractures had healed well after 1 year (Fig. 4). Clinically, at the 1-year follow-up, the patient had excellent range of motion and functional outcomes.

## 3 Discussion

At present, early AC joint fixations are gradually being accepted by many surgeons, especially for complete AC joint injuries (types IV, V, and VI) that involve a high-energy mechanism that results in significant instability of the AC joint. The common methods used to stabilize the AC joint include the Weaver-Dunn procedure, hook plate stabilization, screw stabilization, and anatomic reconstruction of the CC ligaments.^[1–3]^ The only way to prevent malunion in a dislocated midshaft clavicle fracture is with either open reduction internal fixation (ORIF) or a percutaneous procedure.^[4]^ In our case, ORIF was chosen for the left shoulder, and surgical treatment was applied for both type III clavicle fractures. In 2009, Yeh et al^[5]^ reported 1 case with this combination injury. A precontoured plate was used to fix the clavicle. To achieve stability of the clavicle to the acromion and coracoid, a semitendinosus allograft was used to reconstruct the AC and CC ligaments. This was the first report in which ORIF was used for fixation of a clavicle fracture with ipsilateral AC and CC ligament reconstruction. In the present case, we applied a locking hook plate in this rare combination injury. A single implant contoured to stabilize the fractures and AC joint dislocation would have likely provided an ideal construct. However, there was no clavicle hook plate long enough to cover two-thirds of the clavicle length in our inventory. Therefore, we had to cut-off a reconstruction plate and fix the midshaft clavicle fracture and AC joint together with a hook plate. It is unclear whether a stress riser effect will occur between the 2 fixations. At the last follow-up, we found an excellent functional outcome of this fixation and satisfactory progress of the union. To the best of our knowledge, this is the only case in which this particular method has been employed in the management of the described combination injury. Nevertheless, continuous follow-up and biomechanics analysis are needed to support the clinical results and to validate the technique.

In this rare case, which involved simultaneous bilateral midshaft clavicle fractures and unilateral AC joint dislocation, it was important to achieve stability and aim for excellence in terms of full shoulder function. The radiographs and type of injury should be carefully reviewed before treatment. This case also shows that applying surgical treatment to provide a rigid fixation and facilitate good range of movement early and in the long term can result in a good functional outcome.
